# Supplementary figures and images for: Deciphering the immune landscape of head and neck squamous cell carcinoma: A single-cell transcriptomic analysis of regulatory T cell responses to PD-1 blockade therapy
Source: PLoS One. 2023 Dec 14;18(12):e0295863. doi: 10.1371/journal.pone.0295863 (PMC10721039; doi:10.1371/journal.pone.0295863)

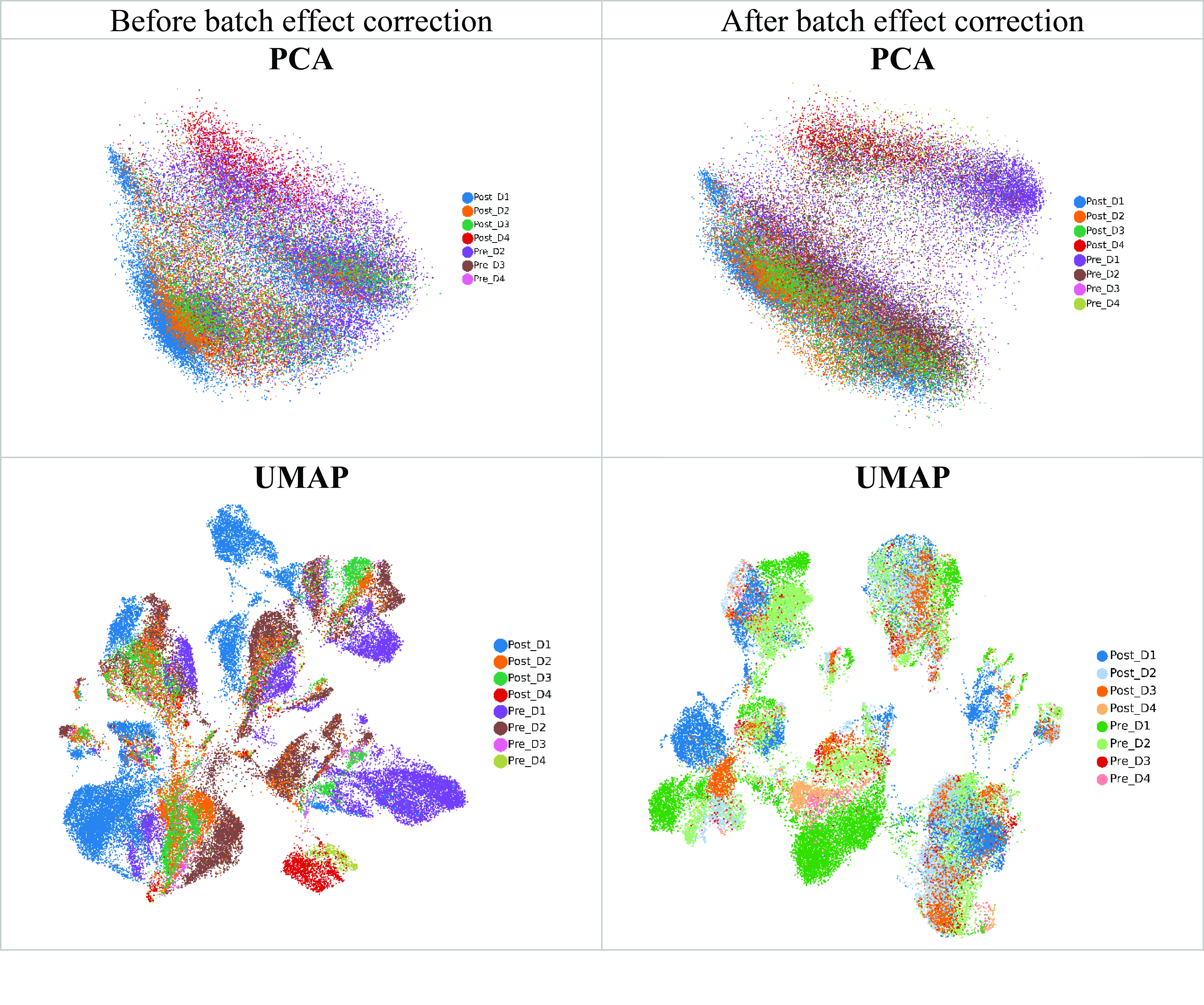

Supplement: S1 Fig — (JPG) [file pone.0295863.s001.jpg]
