# Supplementary material for: Deciphering the immune landscape of head and neck squamous cell carcinoma: A single-cell transcriptomic analysis of regulatory T cell responses to PD-1 blockade therapy
Source: PLoS One. 2023 Dec 14;18(12):e0295863. doi: 10.1371/journal.pone.0295863 (PMC10721039; doi:10.1371/journal.pone.0295863)
Supplement: Attachment — Submitted filename: Response to Reviewers.docx [file pone.0295863.s003.docx]

**Manuscript ID:** PONE-D-23-21092

**Response to the Reviewers**

We thank the two anonymous reviewers for taking the time to carefully assess our manuscript and provide valuable comments and suggestions. We appreciate the opportunity to provide this revised manuscript for further consideration. In this revision, we took care to address each of the concerns raised by the reviewers. In doing so, we believe our manuscript is significantly improved.

**Journal requirements:**

When submitting your revision, we need you to address these additional requirements.

1. Please ensure that your manuscript meets PLOS ONE's style requirements, including those for file naming. The PLOS ONE style templates can be found at https://journals.plos.org/plosone/s/file?id=wjVg/PLOSOne_formatting_sample_main_body.pdf and <https://journals.plos.org/plosone/s/file?id=ba62/PLOSOne_formatting_sample_title_authors_affiliations.pdf>.

Thanks for comment. Done as advised.

2. Please provide Table S1 mentioned in line 356.

Thanks for comment. We removed table S1 as it was not necessary.

3. Thank you for stating the following financial disclosure:

“The funders had no role in study design, data collection and analysis, decision to publish, or preparation of the manuscript.”

At this time, please address the following queries:

a) Please clarify the sources of funding (financial or material support) for your study. List the grants or organizations that supported your study, including funding received from your institution.

b) State what role the funders took in the study. If the funders had no role in your study, please state: “The funders had no role in study design, data collection and analysis, decision to publish, or preparation of the manuscript.”

c) If any authors received a salary from any of your funders, please state which authors and which funders.

d) If you did not receive any funding for this study, please state: “The authors received no specific funding for this work.”

Thanks for the point. Done as advised.

# Funding:

The authors received no specific funding for this work.

Please include your amended statements within your cover letter; we will change the online submission form on your behalf.

Thanks for the comment. Done as advised.

4. In your Data Availability statement, you have not specified where the minimal data set underlying the results described in your manuscript can be found. PLOS defines a study's minimal data set as the underlying data used to reach the conclusions drawn in the manuscript and any additional data required to replicate the reported study findings in their entirety. All PLOS journals require that the minimal data set be made fully available. For more information about our data policy, please see http://journals.plos.org/plosone/s/data-availability.

# Data Availability

The original data which used in this study, can find online through (<https://www.ncbi.nlm.nih.gov/geo/query/acc.cgi?acc=GSE195832>).

The code to reproduce the results is available at <https://github.com/SafarpourLab/HNSCC>.

Upon re-submitting your revised manuscript, please upload your study’s minimal underlying data set as either Supporting Information files or to a stable, public repository and include the relevant URLs, DOIs, or accession numbers within your revised cover letter. For a list of acceptable repositories, please see http://journals.plos.org/plosone/s/data-availability#loc-recommended-repositories. Any potentially identifying patient information must be fully anonymized.

Done as advised. In data availability section, all data and codes which used in this study, was added.

# Data Availability

The original data which used in this study, can find online through (<https://www.ncbi.nlm.nih.gov/geo/query/acc.cgi?acc=GSE195832>).

The code to reproduce the results is available at <https://github.com/SafarpourLab/HNSCC>.

Important: If there are ethical or legal restrictions to sharing your data publicly, please explain these restrictions in detail. Please see our guidelines for more information on what we consider unacceptable restrictions to publicly sharing data: http://journals.plos.org/plosone/s/data-availability#loc-unacceptable-data-access-restrictions. Note that it is not acceptable for the authors to be the sole named individuals responsible for ensuring data access.

None.

We will update your Data Availability statement to reflect the information you provide in your cover letter.

5. We note that Figure 1 in your submission contain copyrighted images. All PLOS content is published under the Creative Commons Attribution License (CC BY 4.0), which means that the manuscript, images, and Supporting Information files will be freely available online, and any third party is permitted to access, download, copy, distribute, and use these materials in any way, even commercially, with proper attribution. For more information, see our copyright guidelines: http://journals.plos.org/plosone/s/licenses-and-copyright.

We require you to either (1) present written permission from the copyright holder to publish these figures specifically under the CC BY 4.0 license, or (2) remove the figures from your submission:

a. You may seek permission from the original copyright holder of Figure 1 to publish the content specifically under the CC BY 4.0 license.

We recommend that you contact the original copyright holder with the Content Permission Form (http://journals.plos.org/plosone/s/file?id=7c09/content-permission-form.pdf) and the following text:

“I request permission for the open-access journal PLOS ONE to publish XXX under the Creative Commons Attribution License (CCAL) CC BY 4.0 (http://creativecommons.org/licenses/by/4.0/). Please be aware that this license allows unrestricted use and distribution, even commercially, by third parties. Please reply and provide explicit written permission to publish XXX under a CC BY license and complete the attached form.”

Thanks for the comment. We have mentioned we used Biorender in revised manuscript and also, we got permission license from the related study (License Number:5635221448503).

Please upload the completed Content Permission Form or other proof of granted permissions as an "Other" file with your submission.

In the figure caption of the copyrighted figure, please include the following text: “Reprinted from [ref] under a CC BY license, with permission from [name of publisher], original copyright [original copyright year].”

Thanks for the comment. Done as advised.

b. If you are unable to obtain permission from the original copyright holder to publish these figures under the CC BY 4.0 license or if the copyright holder’s requirements are incompatible with the CC BY 4.0 license, please either i) remove the figure or ii) supply a replacement figure that complies with the CC BY 4.0 license. Please check copyright information on all replacement figures and update the figure caption with source information. If applicable, please specify in the figure caption text when a figure is similar but not identical to the original image and is therefore for illustrative purposes only.

[Note: HTML markup is below. Please do not edit.].

**Reviewer #1:**

This paper presents a study to investigate the effect of Nivolumab on the function and cellular communication of Tregs using scRNA-seq data analysis. Authors gained a better understanding of the mechanisms underlying the response and resistance to anti-PD-1 CPI in HNSCC. This study had the potential to contribute to the development of more effective immunotherapeutic strategies for the treatment of HNSCC. However, there are questions that limit my enthusiasm of the paper, as outlined below.

1- How can authors generalize the findings based on using only one scRNA-seq data (GSE195832)? Any validation study to assess the performance of findings.

Thanks for the comment. Our study focused on HNSCC, we have indeed conducted a comprehensive search of available databases up to the current date, September 24, 2023, to identify additional datasets that could complement our analysis. Regrettably, we encountered significant challenges in identifying a suitable dataset that aligns with our study's objectives and design. HNSCC is known for its remarkable heterogeneity, particularly across different sub-sites, which makes it challenging to find datasets that are directly comparable to our own.

We did, however, locate a single dataset (GSE225170) that bears some similarity, although not identical, to our research focus. This dataset pertains to HNSCC but involves the treatment of pembrolizumab (an alternative Anti-PD1 agent). Based on celltypist automated annotation, it contains a relatively small number of cells, specifically only 9115, which limits its reliability for downstream analysis. Moreover, it notably lacks a sufficient representation of Treg cells, with only 13 cells present. This paucity of Treg cells diminishes its suitability as a validation dataset for our specific study, given our emphasis on Treg cell dynamics.

2- In data section, add more details about the TCGA data and normalized expression data (e.g., TPM).

Thanks for the point. In order to meet the reviewer comment, we have updated the figure 2 caption as below:

The box plots display the median mRNA expression levels of Treg markers in HNSCC tumors (orange) compared to normal tissues (red). The values on the axes are presented in Log2 (TPM+1), where TPM (Transcripts Per Million) quantifies gene expression while considering transcript length and sequencing depth. Log2 transformation makes the data more interpretable and robust for visual comparisons. The "+1" avoids issues with zero TPM values, ensuring all values are positive.

3- Authors explain the PCA and combat analyses to remove potential variation or batches. Please add the plots and more details as supplementary file. More visualization plots related to before and after correction (using top 20 PCS!!). How are the technical variations associated with age and sex?

Thank you for bringing this to our attention. We included PCA plots in our supplementary to visually demonstrate the initial integration patterns. It's important to clarify that for the PCA, we utilized the default setting of n_comps=50, which was incorrectly stated in the initial draft. As shown in below figure, combat could successfully reduce the batch effect of samples.

It's worth mentioning that the original dataset, GSE195832, did not provide information on the age and sex of the samples, which regrettably limits our ability to directly investigate any potential links between technical variations and these demographic factors within the scope of this specific study.

| Before batch effect correction | After batch effect correction |
| --- | --- |
| **PCA** | **PCA** |
| **UMAP** | **UMAP**  |

4- For the scRNAseq data, authors only mentioned normalization approach, while need more details about pre-processing steps.

Thank you for pointing that. In revised version of manuscript, we provided more details regarding to preprocessing step as below:

In the analysis of scRNA-seq data using Scanpy (version 1.9.1), a rigorous pipeline was meticulously executed. Initially, a quality control step identified and eliminated low-quality cells with less than 200 expressed genes and those with more than 20% mitochondrial content. Additionally, genes expressed in fewer than 20 cells were filtered out. Subsequent data preprocessing involved the normalization of raw gene expression counts using the *sc.pp.normalize_total* function, with a target total sum of 10,000 counts per cell, and a logarithmic transformation to stabilize variance. To capture the most informative genes, Highly Variable Genes (HVGs) were precisely selected using the *sc.pp.highly_variable_genes* function, and retaining the top 4000 genes. These HVGs were then subjected to principal component analysis (PCA) [1] to reduce dimensionality. To address batch effects, the 'combat' algorithm (version 0.3.3) [2] was meticulously applied to harmonize PCA embeddings from eight distinct samples. Utilizing the top 50 principal components, a neighborhood graph was constructed to capture cell-cell similarities (Fig. S1), and the Leiden algorithm was precisely utilized for clustering cells into biologically meaningful groups. Finally, differential expression analysis, leveraging the *sc.tl.rank_genes_group* function with use_raw=True, was conducted to uncover genes with significant expression differences within each cluster.

5- Enrichment analysis using two different web-apps. The Enrichr web-app is based on ORA while the other one was mentioned on line 390 also included the ORA. Please clarify this part. Be more precise about where or for which analysis the GSEA or ORA were applied.

Thank you for your comment. We used both Gene Set Enrichment Analysis (GSEA) and Over-Representation Analysis (ORA) techniques in our study. Specifically, we utilized Enrichr for ORA and WebGestalt for GSEA to enhance the reliability of our findings. By combining these methods, we aimed to provide a more comprehensive evaluation of our research hypotheses. We've revised our manuscript to clarify the specific contexts in which ORA and GSEA were applied, addressing the reviewer's feedback for improved transparency and clarity in our methodology.

In our study, we performed gene enrichment analysis using two distinct methods to comprehensively evaluate the biological implications of differentially expressed genes (DEGs) within the Tregs subpopulation. First, we employed Over-Representation Analysis (ORA) with Enrichr (https://maayanlab.cloud/Enrichr/), which allowed us to assess whether the DEGs were significantly associated with specific Gene Ontology (GO) terms of biological processes. This approach highlighted over-represented functional categories among the DEGs. Additionally, we utilized Gene Set Enrichment Analysis (GSEA) with WebGestalt (http://www.webgestalt.org/option.php), a robust tool that assessed whether our DEGs exhibited coordinated and statistically significant expression patterns within predefined gene sets from the Molecular Signature Database (MSigDB). This dual approach ensured a comprehensive exploration of the functional relevance of the DEGs, providing valuable insights into the underlying biological processes and pathways within the Tregs subpopulation.

6- Authors mentioned different web-app, EnrichR, GEPIA database, SquidPy, scFates, CellPhone DB, Pseudo-time trajectory analysis, etc., while not enough details were shared about these web-apps including methods briefly, etc. Not easy to follow to readers.

Thanks for the point. Done as advised.

In our study, we performed gene enrichment analysis using two distinct methods to comprehensively evaluate the biological implications of differentially expressed genes (DEGs) within the Tregs subpopulation. First, we employed Over-Representation Analysis (ORA) with Enrichr (https://maayanlab.cloud/Enrichr/), which allowed us to assess whether the DEGs were significantly associated with specific Gene Ontology (GO) terms of biological processes. This approach highlighted over-represented functional categories among the DEGs. Additionally, we utilized Gene Set Enrichment Analysis (GSEA) with WebGestalt (http://www.webgestalt.org/option.php), a robust tool that assessed whether our DEGs exhibited coordinated and statistically significant expression patterns within predefined gene sets from the Molecular Signature Database (MSigDB). This dual approach ensured a comprehensive exploration of the functional relevance of the DEGs, providing valuable insights into the underlying biological processes and pathways within the Tregs subpopulation.

We conducted pseudotime analysis using scFates v0.8.1 (https://pypi.org/project/scFates/), an analytical tool seamlessly integrated with Scanpy and notable for its GPU-accelerated capabilities, facilitating faster and more scalable inference. Pseudotime analysis involves the estimation of cellular progression along developmental trajectories and examination of gene expression pattern across pseudotime, and scFates is well-suited for this purpose. It allowed us to infer pseudotime values for individual cells, providing insights into their developmental states. By comparing these pseudotime genes with DEGs, we identified Treg-specific genes within the trajectory. This approach helped unravel the temporal dynamics and critical cellular transitions within the biological system under investigation.

CellPhoneDB is particularly notable for its ability to handle a substantial number of interacting cell pairs, often exceeding 100,000, and it accommodates the analysis of interactions across diverse cluster combinations, frequently numbering over 100 clusters. To ensure the reliability of our findings, we rigorously considered interactions where both ligand and receptor genes were expressed in at least 10% of the cells within our scRNA-seq dataset.

GEPIA, a user-friendly web-based tool designed for the analysis and visualization of gene expression pattern across multiple cancers from the Cancer Genome Atlas (TCGA) and the Genotype-Tissue Expression (GTEx) projects.

7- Which software was used? Any codes to be shared through GitHub?

The software used for the analysis pipeline in this study was Scanpy version 1.9.1. The specific codes used in the study are available on GitHub at the following repository: <https://github.com/SafarpourLab/HNSCC>.

# Data Availability

The code to reproduce the results is available at <https://github.com/SafarpourLab/HNSCC>.

8- How the canonical gene markers were selected (CD3D, FOXP3, TIGIT, and FANK1)?

Thanks for the comment. We have annotated Tregs based on literature markers which reported in previous studies [3, 4]. We have mentioned the references in revised manuscript.

The regulatory T cells (T_regs_) were annotated with specific markers such as *CD3D*, *FOXP3*, *TIGIT*, and *FANK1* which reported in previous studies [3, 4].

9- Figure 2D add the y-axis label. What is the statistical method to compare two groups (N vs T)? Figure 2G needs to re-generate. There IS NOT Figure 2H.

Thank you for your feedback. In Figure 2D, we have added the y-axis label for clarity. Regarding the statistical method used to compare the two groups (N vs. T), GEPIA employed a One-way ANOVA using disease state (Tumor or Normal) as a variable to calculate differential expression. Additionally, we have updated the labels in Figure 2D as per your suggestion. Regarding Figure 2G, we generated it to ensure accuracy and completeness. As for Figure 2H, please note that it does not exist in our study and in revised manuscript, we corrected the issue.

10- DEG analysis: There are some questions about

a. Is the t-test paired or two group independent?

Thank you. We used paired t-test method for DEG analysis. In revised manuscript we mentioned that in related section.

b. Fig 3A does not make sense. Why there are some outliers (log FC > 10 or < -10)?

Thank you for highlighting the issue.

In our initial analysis, we used an adjusted p-value threshold of ≤0.05 for identifying Differentially Expressed Genes (DEGs). Regrettably, in Figure 2A, this threshold was incorrectly labeled as 'p-value.' In the revised version, we have rectified this labeling error for clarity and accuracy.

On the other hand, extreme outliers can arise from various sources, such as technical artifacts, exceptional biological variations in gene expression, or potential issues during data preprocessing. In instances where such extreme log fold changes are coupled with remarkably low adjusted p-values, it signifies that these genes exhibit exceptionally significant expression differences between the compared groups, even if their expression levels may seem atypical. In such cases, it is often prudent to retain these genes in the analysis, as they might hold substantial biological relevance.

In our study, based on an adjusted p-value threshold of 0.05, we detected a total of 75 outliers. Intriguingly, it's noteworthy that approximately half of these outliers exhibit adjusted p-values lower than 0.01. This observation underscores the complexity of our dataset and suggests that if we were to employ a primary filter stringent enough to eliminate all outliers, we would inadvertently exclude numerous potentially significant genes from our analysis. As a result, we have made the deliberate decision not to remove these outliers, ensuring that we encompass and evaluate genes that could hold substantial importance in our study.

Plot is confusing any cut-off for log FC? It is not clear on figure the legend.

Thank you for raising this point. As previously detailed in the DEG analysis section, genes were identified as DEGs when they satisfied the following criteria: an adjusted p-value of < 0.05 and an log_2_FC >0. In response to the reviewer's feedback, we have duly revised the caption for Figure 3 and provided a clarifying explanation for the FC cutoff to enhance the clarity and comprehensibility of our findings.

Why authors chose the DEGs based on p-value and not the FDR or corrected p-value?

Thank you for highlighting the issue.

In our initial analysis, we used an adjusted p-value threshold of ≤0.05 for identifying Differentially Expressed Genes (DEGs). Regrettably, in Figure 2A, this threshold was incorrectly labeled as 'p-value.' In the revised version, we have rectified this labeling error for clarity and accuracy.

11- Fig 3B is based on ORA analyses? Add at the caption. Fig 3D, what is the y-axis? Why the G1 has not defined on caption. More precise about p-value or FDR (corrected p-value). The DEG or pathway results should be based on FDR.

Thank you for your feedback. We have taken your suggestions into account and made the necessary revisions to the manuscript as follows:

**Fig 3B Caption**: We have updated the caption for Fig 3B to explicitly state that it is based on Over-Representation Analysis (ORA) analyses.

**B)** GO Biological Processes of Treg’s DEGs using ORA.

**Fig 3D Y-Axis Label**: The y-axis of Fig 3D represents the percentage of total Treg cells in pre and post nivolumab treatment across different cell cycle phases. We have also added this information to the figure for clarity.

**G1 Label**: In response to your suggestion, we have included a clear description of the G1 phase in the revised manuscript.

(G1: Growth 1 phase, S: DNA Synthesis phase, G2M: Checkpoint, Mitosis phase) and statues.

**p-value and FDR**: We have reported the DEGs based on adjusted p-values and have explicitly mentioned this in the manuscript. Additionally, for pathway analysis, we utilize the EnrichR Appyter, which typically visualizes the top ten enriched terms along with their p-values. In the revised manuscript, we have clarified that we report both DEGs and pathway analysis results (both GSEA and ORA) based on p-values.

12- Fig 4A, what do the rows and columns represent? why not clustering for genes? the color bar is missing. How the top 10 genes (up-regulating selected). It is not clear from heatmap.

Thank you for your feedback. In Figure 4A, the UMAP and tree diagram represent the different fates of the HNSCC TME. The trajectory starts from the 0 branch and ends on the 1 branch, illustrating the progression of TME states. To clarify the representation and address the reviewer's concerns, we have revised the figure to ensure it is more intuitive and less confusing.

The heatmap columns in Figure 4A correspond to the transitions from branch 0 to branch 1, and the rows represent all of the genes that are up-regulated in Tregs across these different fates. To identify the top ten genes among these up-regulated genes, we have utilized the default highlighting method in our analysis software (scFates). (https://scfates.readthedocs.io/en/latest/Basic_Curved_trajectory_analysis.html#Preparing-the-environment-for-the-tutorial)

To enhance clarity, we have made revisions to the figure and the accompanying manuscript text.

Fig. 4:

UMAP presentation illustrating the dynamics of the HNSCC TME and its transition states as they progress through pseudo-time. The lineage tracking initiates at the 0 branch and concludes at the 1 branch. The heatmap depicts genes that experience upregulation within the Treg branch as pseudo-time advances. The highlighted genes comprise the top ten upregulated genes specific to the Treg branch compared to other branches during pseudo-time analysis.

13- Fig 4c, I assume the y-axis represent the log-expression transformed. If yes, why here is -1 or less than zero?

Thank you for your feedback. In our analysis using scFates, we designated a specific gene as the root (scf.tl.root(adata, "gene name")). In this instance, we chose the MARCO gene as the root because it exhibited the highest expression within branch 0, which corresponds to TAMs. However, MARCO had relatively low expression in the other cells across various branches. Consequently, the expression of the gene of interest is compared to MARCO as the baseline (with a baseline expression level of 0). It's possible that within the different branches, a few cells express MARCO at a low level, resulting in negative values for our gene of interest. The peak you see in the graph corresponds to the Treg branch, and as expected, our gene of interest is highly expressed in this context.

14- Figure 5, same comments as Figures 2 and 4.

Thanks for the comment. Done as advised. The below explanation was added. Furthermore, we added the scale-bar to each plot.

**D)** The expression pattern of significant IICs along the pseudo-time; Yellow branches shows highest expression of the gene across the pseudo-time.

15- How was the cell-cell interaction assessed? Any metrics or visualization plot to show how the 5 pairs were selected? Did you measure the correlation across ligand-receptor pairs? The enrichment analysis (Fig 6D) was applied based on the 10 genes? Please add more details about the enrichment analysis.

Thank you for your comment. As mentioned in M&M section (Cell-cell communication part), to assess cell-cell interactions, we utilized Squidpy, which is a re-implementation of the well-established CellphoneDB method for ligand-receptor interaction analysis. This method employs a permutation-based test to evaluate the expression of ligands and receptors across various cell-type combinations. Specifically, it calculates the mean expression of both the ligand and receptor between cell types and establishes a null distribution through n permutations (defaulted to 1,000). Subsequently, a P-value is computed based on the proportion of permuted means relative to the actual mean. In the case of ligands or receptors composed of multiple subunits, the test considers the minimum expression.

The selection of the top 5 pairs was performed by comparing the list of ligand-receptor interaction of Tregs and TAMs. Notably, these top 5 pairs were found to be associated with DEGs in either TAMs or Tregs, which has been explicitly stated in the manuscript.

In response to the reviewer's suggestion, we have included a detailed description of the enrichment analysis and the metrics employed for the selection of the top 5 pairs in the revised manuscript.

We identified hallmark associations for these ligands and receptors using MSigDB, focusing on the top 5 pairs (10 genes). The analysis was conducted using ORA and revealed connections to various cellular pathways, including those related to inflammation response, apoptosis, glycolysis, angiogenesis, and signaling pathways such as IL-6/JAK/STAT3 signaling, IL2/STAT5 signaling, and TNFα signaling via NF-κB (Fig. 6D).

16- All the abbreviations should be defined in advance (e.g., TAM).

Thanks for the comment. Done as advised.

# Abbreviations:

HNSCC, Head and Neck Squamous Cell Carcinoma; TME, Tumor Microenvironment; scRNA-seq, Single-Cell RNA-sequencing; Treg, Regulatory T cells; CPI, Anti-PD-1 Checkpoint Inhibitors; TAMs, Tumor Associated Macrophages; TCGA, The Cancer Genome Atlas; MSigDB, Molecular Signature Database

**Reviewer #2:**

The authors have re-analysed the published scRNAseq data. The manuscript submitted is technically sound and well written. Statical analysis are appropriately done and the given data support their conclusions. However, if accepted for publication, the clarity of the figures should be improved (high resolution figures). The figure markings are not visible properly in the present form.

Thanks for the comment. In first round of revision, we have uploaded the figures with high quality resolution separately.

1.	Wolf, F.A., P. Angerer, and F.J. Theis, *SCANPY: large-scale single-cell gene expression data analysis.* Genome biology, 2018. **19**(1): p. 1-5.

2.	Zhang, Y., G. Parmigiani, and W.E. Johnson, *ComBat-seq: batch effect adjustment for RNA-seq count data.* NAR genomics and bioinformatics, 2020. **2**(3): p. lqaa078.

3.	Chen, K., et al., *Single-cell RNA-seq reveals dynamic change in tumor microenvironment during pancreatic ductal adenocarcinoma malignant progression.* EBioMedicine, 2021. **66**.

4.	Zhao, J., et al., *Single-cell RNA sequencing reveals the heterogeneity of liver-resident immune cells in human.* Cell Discovery, 2020. **6**(1): p. 22.
